# Three-Dimensional Graph Matching to Identify Secondary Structure Correspondence of Medium-Resolution Cryo-EM Density Maps

**DOI:** 10.3390/biom11121773

**Published:** 2021-11-26

**Authors:** Bahareh Behkamal, Mahmoud Naghibzadeh, Mohammad Reza Saberi, Zeinab Amiri Tehranizadeh, Andrea Pagnani, Kamal Al Nasr

**Affiliations:** 1 Department of Computer Engineering, Faculty of Engineering, Ferdowsi University of Mashhad, Mashhad 9177948974, Iran; b.behkamal@mail.um.ac.ir; 2 Medicinal Chemistry Department, School of Pharmacy, Mashhad University of Medical Sciences, Mashhad 9177899191, Iran; saberiMR@mums.ac.ir (M.R.S.); amiritz@mums.ac.ir (Z.A.T.); 3 Bioinformatics Research Group, Mashhad University of Medical Sciences, Mashhad 9177899191, Iran; 4 Politecnico di Torino, Corso Duca degli Abruzzi 24, I-10129 Torino, Italy; andrea.pagnani@polito.it; 5 Italian Institute for Genomic Medicine, IRCCS Candiolo, SP-142, I-10060 Candiolo, Italy; 6 INFN, Sezione di Torino, I-10125 Torino, Italy; 7 Department of Computer Science, Tennessee State University, Nashville, TN 37209, USA

**Keywords:** protein, cryo-electron microscopy, modeled structure, secondary structure elements, 3D vector matching, 3D graph matching, similarity-based voting algorithm

## Abstract

Cryo-electron microscopy (cryo-EM) is a structural technique that has played a significant role in protein structure determination in recent years. Compared to the traditional methods of X-ray crystallography and NMR spectroscopy, cryo-EM is capable of producing images of much larger protein complexes. However, cryo-EM reconstructions are limited to medium-resolution (~4–10 Å) for some cases. At this resolution range, a cryo-EM density map can hardly be used to directly determine the structure of proteins at atomic level resolutions, or even at their amino acid residue backbones. At such a resolution, only the position and orientation of secondary structure elements (SSEs) such as α-helices and β-sheets are observable. Consequently, finding the mapping of the secondary structures of the modeled structure (SSEs-A) to the cryo-EM map (SSEs-C) is one of the primary concerns in cryo-EM modeling. To address this issue, this study proposes a novel automatic computational method to identify SSEs correspondence in three-dimensional (3D) space. Initially, through a modeling of the target sequence with the aid of extracting highly reliable features from a generated 3D model and map, the SSEs matching problem is formulated as a 3D vector matching problem. Afterward, the 3D vector matching problem is transformed into a 3D graph matching problem. Finally, a similarity-based voting algorithm combined with the principle of least conflict (PLC) concept is developed to obtain the SSEs correspondence. To evaluate the accuracy of the method, a testing set of 25 experimental and simulated maps with a maximum of 65 SSEs is selected. Comparative studies are also conducted to demonstrate the superiority of the proposed method over some state-of-the-art techniques. The results demonstrate that the method is efficient, robust, and works well in the presence of errors in the predicted secondary structures of the cryo-EM images.

## 1. Introduction 

Proteins are one of the essential parts of all organisms that perform most of the tasks of living species. To study the relationship between protein structure and function, it is necessary to have access to precise three-dimensional (3D) structural information [1]. Hence, understanding the protein structure is of great interest to biologists. Traditionally, protein structures have been obtained using experimental techniques such as X-ray crystallography and NMR spectroscopy. X-ray crystallography has been used to study thousands of protein complexes which are crystallizable. NMR spectroscopy is limited to small molecules of an atomic mass less than 50 kDa. Therefore, neither of these techniques can be used to study molecular complexes which can be found in nature in their near-native state [2]. More recently, cryo-electron microscopy (cryo-EM) has emerged as an experimental technique to address most of the scalability concerns of the traditional techniques by being able to image large macromolecular complexes, such as ribosomes and viruses, in their native conformations. This widely used technique does not require crystalizing before data acquisition and it is applicable on a molecule larger than ~100 kDa [3,4]. In recent years, there have been significant advances in cryo-EM imaging techniques [5]. However, for some cases, the cryo-EM reconstructions are limited to medium-resolution (~4–10 Å), where the secondary structure elements can be computationally and visually identified, but not the individual amino acid residues [6]. This lack of atomic-level resolution leads to many computational challenges for protein 3D structure determination. For the density maps at high-resolution (~2–4 Å), the backbone is recognizable, and the protein structure at the atomic level can be directly derived. However, for the low (~10–25 Å) or medium-resolution (~4–10 Å) density maps, the backbone of the protein and the atomic information cannot be directly achieved from the cryo-EM maps. This limitation has motivated the development of many computational methods that use the medium-resolution cryo-EM map to collect protein structural information [7,8,9,10,11,12,13,14,15]. In the cryo-EM modeling pipeline, some major steps should be handled, such as extracting the secondary structure elements on a cryo-EM density map and matching them to a sequence/model, the $C_{\propto }$ placement of SSEs, building an atomic structure, and structure optimization [6]. One of the main challenging and critical steps is finding the mapping of the secondary structures of the modeled structure to the cryo-EM map. This is because this step provides the initial anchor point to find the location of the $C_{\propto }$ atoms and to construct the protein backbone. The precise identification of SSEs correspondence enables us to produce an accurate initial 3D structure of a protein that can be refined further by later steps in the model-building pipeline. 

At medium-resolution, the analyses of cryo-EM maps rely on the availability of the known protein structures obtained by other high-resolution experimental methods (X-ray crystallography, NMR). When the atomic structure from other sources of information is not accessible, a de novo modeling approach could be utilized [9,16,17,18,19,20]. S. Abeysingh et al. [16] introduced a research study on solving the α-helix correspondence problem through shape matching by modeling both a 1D sequence and a 3D volume to attributed relational graphs. Furthermore, they developed Gorgon [21], which is an interactive molecular modeling toolkit with an interactive visualization platform. Al Nasr et al. developed a weighted directed graph to solve the secondary structure assignment and presented an approach to enumerate the top-ranked topologies instead of enumerating all possible topologies [18]. The authors conducted another study, DP-TOSS, to solve the topology determination based on a layered graph using a dynamic programming approach into a constrained k-shortest path algorithm [19]. DP-TOSS was compared with Gorgon in our previous study [19]. The results indicated that DP-TOSS was superior to Gorgon. Afterwards, Biswas et al. [22] enhanced the performance of DP-TOSS by combining the information from multiple secondary structure prediction servers. They utilized some different structural information, such as the length of secondary structures, the loop length, and the skeleton between two secondary structure traces as a scoring function. Al Nasr et al. enhanced the DP-TOSS accuracy using the efficient scoring methodology. The proposed scoring functions were a skeleton-based scoring function, a geometry-based function, and a multi-well potential energy-based function [20]. 

In the presence of a high-resolution structure for an insufficient resolution cryo-EM map, the fitting methods, which are categorized into flexible and rigid-body fitting, could be utilized to derive the atomic structure from the cryo-EM map [9,12,14,17]. Early studies have concentrated on searching for the optimal position and orientation of a protein’s secondary structure components with the best overlaps with the SSEs extracted from a cryo-EM density map [23,24,25,26]. Dou et al. proposed a flexible fitting of an atomic structure into a cryo-EM map which is guided by the correspondences between α-helices in the atomic model and the cryo-EM map [27]. In the work of [28], a computational method is presented to quantify the agreement between two sets of central axes of α-helices which are relevant to atomic structures and cryo-EM maps. It utilized an arc-length association strategy to characterize the lateral and the longitudinal differences of the two axes. 

Our approach in this study is to introduce a novel geometrical matching approach to find the correct matches between SSEs-C and SSEs-A (SSEs correspondence). The central theme of our approach is to cast the SSEs mapping problem as that of three-dimensional graph matching. For this purpose, the SSEs matching problem is formulated as a 3D vector matching problem in Cartesian coordinate space. Then, the 3D vector matching problem is transformed into a 3D graph matching problem. To solve the 3D graph matching problem, three novel mathematical-based features, as well as two robust statistical scoring functions, are proposed. Finally, to obtain the final SSEs assignment among all possible ones, a similarity-based voting algorithm combined with the PLC concept is developed. Furthermore, the results show the superiority of the proposed method compared to some of the state-of-the-art techniques.

## 2. Materials and Methods

**Figure 1:**
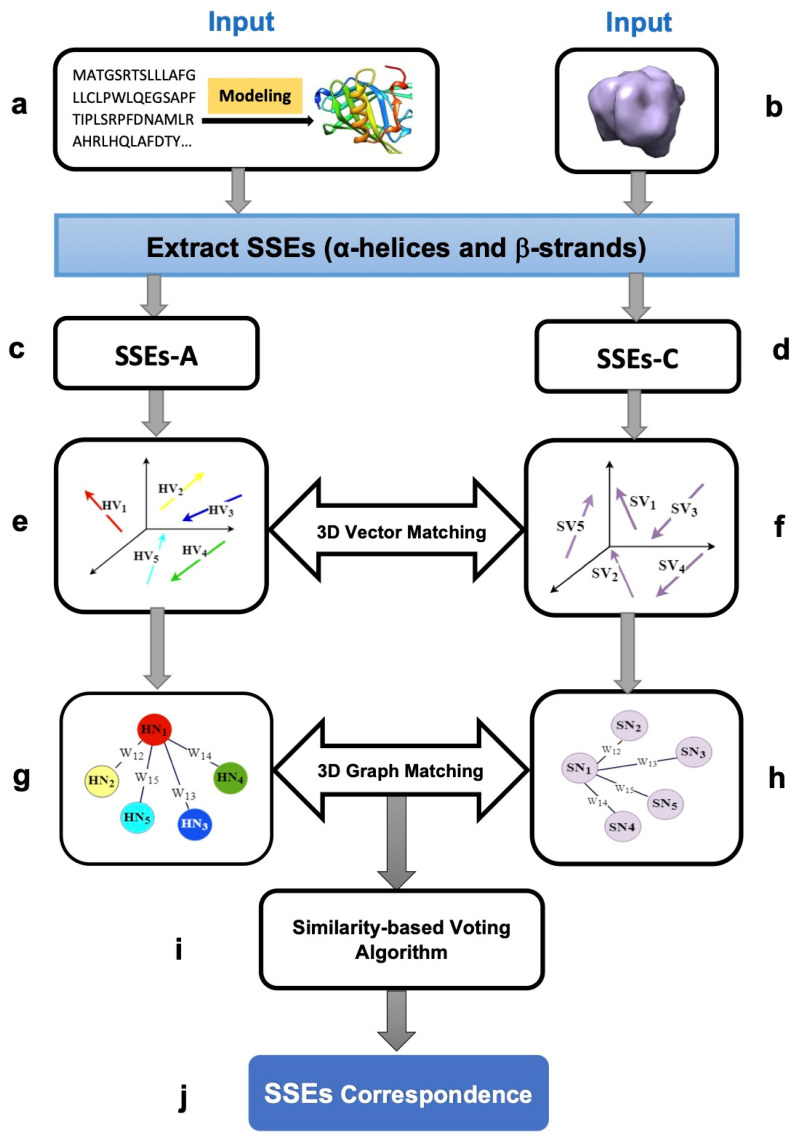
Different stages of the framework pipeline: (**a**) the inputs, including the modeled structure (PDB ID: 1BJ7, chain A) visualized by Chimera [29]; (**b**) the density map simulated at 10 Å resolution using protein structure 1BJ7 and Chimera package [29]; (**c**) the secondary structure elements extracted from the 3D modeled structure in the preprocessing step (SSEs-A); (**d**) the secondary structure elements extracted from the cryo-EM density map (SSEs-C); (**e**) the 3D vectors constructed based on the extracted SSEs-A; (**f**) the 3D vectors constructed based on the extracted SSEs-C; (**g**,**h**) the 3D graphs are constructed; (**i**) the similarity-based voting algorithm is proposed as a decision making strategy for finding the SSEs correspondence; (**j**) the secondary structure elements correspondence.

In this section, an automatic assignment method for finding the SSEs correspondence in three-dimensional space is proposed. An overview of the method is illustrated in Figure 1. The method takes the modeled structure and the medium-resolution cryo-EM density map as inputs (Figure 1a,b) and produces SSEs correspondence as output. Initially, in the preprocessing step, the α-helices and β-strands from the modeled structure (SSEs-A) and the cryo-EM map (SSEs-C) are extracted (Figure 1c,d). Then, the extracted SSEs from both the structure and the map are constructed as vectors in the three-dimensional Cartesian coordinate systems (Figure 1e,f). After that, utilizing the novel strategy and innovative mathematical-based features (i.e., angle, Euclidian distance, and relative length), the 3D vector matching problem is transformed into the 3D graph matching problem (Figure 1g,h). To solve the 3D graph matching problem, two robust statistical scoring functions, which are Bhattacharyya distance (BD) and modal assurance criterion (MAC), are proposed. At the end, a similarity-based voting algorithm has been developed (Figure 1i) to extract the SSEs correspondence.

### 2.1. Preprocessing

In this step, the model, generated by I-TASSER [30,31,32,33], and the cryo-EM density map are used as initial inputs and the geometrical features are returned as outputs. Generally, the protein modeling can be performed using various modeling tools such as Modeller [34], AlphaFold [35,36], RaptorX [37,38,39], and I-TASSER. I-TASSER (Zhang-Server) and AlphaFold (A7D) are two efficient and robust methods, which are based on deep residual-convolutional networks. AlphaFold utilizes artificial intelligence and deep learning methods to generate the 3D structure of proteins. The framework of the AlphaFold is based on a deep two-dimensional convolutional residual network that enables this method to create high-accuracy structures even under sequences with fewer homologous sequences. I-TASSER is developed for automated protein structure prediction, which performs the model construction by collecting the high-scoring structural templates based on the threading approaches. The hierarchical architecture is composed of four steps, including threading, structural assembly, model selection, and structure-based functional annotation. I-TASSER finds a protein template of similar super-secondary structures from the Protein Data Bank (PDB) through LOMETS [40,41]. Then, the extracted segments from the templates are reconstructed through replica-exchange Monte Carlo simulations. The performance of the generated model is assessed based on the reliability of the threading templates and the convergence parameters of the structural assembly. The server was successful in the Critical Assessment of Techniques for Protein Structure Prediction (CASP) competition in recent years. Hence, in this study, the authors opted for I-TASSER, which is available at (https://zhanggroup.org/I-TASSER/, accessed on 30 September 2021) due to its simplicity and high accuracy. 

**Figure 2:**
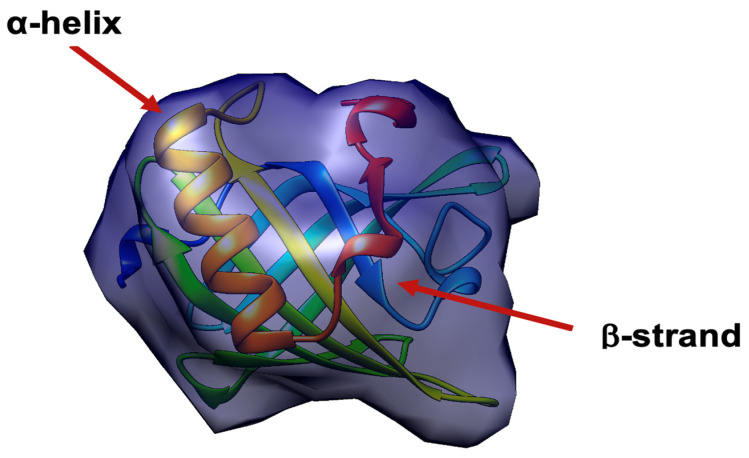
Secondary structure elements (α-helices and β-strands) in the fitted atomic structure with cryo-EM map visualized by Chimera [29].

The geometrical features are Cartesian coordinate voxels of the SSEs (α-helices and β-strands). For more clarification, the α-helices and β-strands are the primary elements of the secondary structures, as illustrated in Figure 2. These elements are formed by amino acid residues. Each residue consists of four primary atoms (N, $C_{\propto }$, C, and O). The $C_{\propto }$. atom is the most important one in the backbone of the SSEs. For the first input (i.e., the 3D model), all the $C_{\propto }$ coordinates of the SSEs-A (the geometrical location of the backbone alpha carbon of the α-helices and β-strands) are extracted. The second input is the cryo-EM map. At a medium-resolution cryo-EM map, the secondary structure components can be observed as density rods [17]. Various computational methods, such as SSEhunter [42], SSELearner [43], SSETracer [44], and Emap2sec [45] have been developed to detect the position, orientation, and length of α-helices and β-strands on the cryo-EM images. In this study, the Cartesian coordinate voxels of the SSEs-C have been extracted using SSETracer [44]. 

### 2.2. Construction of 3D Vectors from SSEs-A and SSEs-C

**Figure 3:**
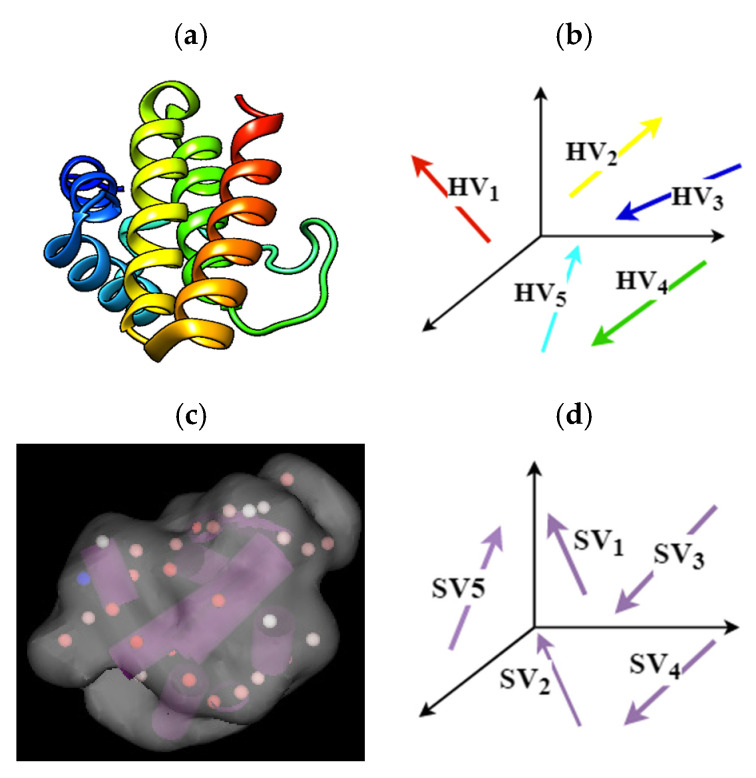
Construction of 3D vectors from extracted SSEs: (**a**) 3D structure of protein 1FLP (PDB ID) is shown with chimera [29]; (**b**) each α-helix in the atomic model is considered as a helix vector (HV) in the Cartesian coordinate system ($R_{\mathrm{SSEs}-A}^{3}$); (**c**) the cryo-EM density map and the SSEs-C detected on it. The map is simulated at 10 Å resolution using protein structure 1FLP (PDB ID). The location of SSEs-C is illustrated as purple cylinders with Gorgon [21]; (**d**) extracted SSEs-C on the map considered as stick vector (SV) in three-dimensional Cartesian space $R_{\mathrm{SSEs}-C}^{3}$.

This study aims to find the correspondence between the α-helices and β-strands detected on the cryo-EM map with those extracted on the modeled structure. To deal with this issue, the extracted SSEs from the map and the 3D model are converted to the 3D vectors in the Cartesian coordinate system. For visualization, a simple α-protein 1FLP (PDB ID) is selected from the data set of interest, as demonstrated in Figure 3. The start and end voxels of the SSEs-A have been utilized to construct the 3D vectors (Figure 3a,b). Since we do not have any information regarding the $C_{\propto }$ atom of the medium-resolution cryo-EM map, the coordinate voxels of the central axis of the SSEs-C have been used to construct the 3D vectors (Figure 3c,d). 

### 2.3. Three-Dimensional Vector Matching 

In order to solve the vector matching problem, three effective mathematical-based features, which are the angle, the Euclidean distance, and the relative length, are proposed. These features are computed with the aid of all vectors in $R_{\mathrm{SSEs}-A}^{3}$. and $R_{\mathrm{SSEs}-C}^{3}$. Afterward, the 3D vector matching problem is transformed into the 3D graph matching problem based on the extracted features. The construction of the graph is elaborated in the following.

#### Construction of Weighted Fully Connected Graphs of SSEs-A and SSEs-C

Based on the problem at hand, the central idea of the method is to find the correspondence between the constructed 3D vectors of $R_{\mathrm{SSEs}-A}^{3}$ and $R_{\mathrm{SSEs}-C}^{3}$. Hence, two weighted fully connected graphs (i.e., $G_{\mathrm{SSEs}-A}$. and $G_{\mathrm{SSEs}-C}$) have been constructed from $R_{\mathrm{SSEs}-A}^{3}$ and $R_{\mathrm{SSEs}-C\;}^{3}$. 

**Figure 4:**
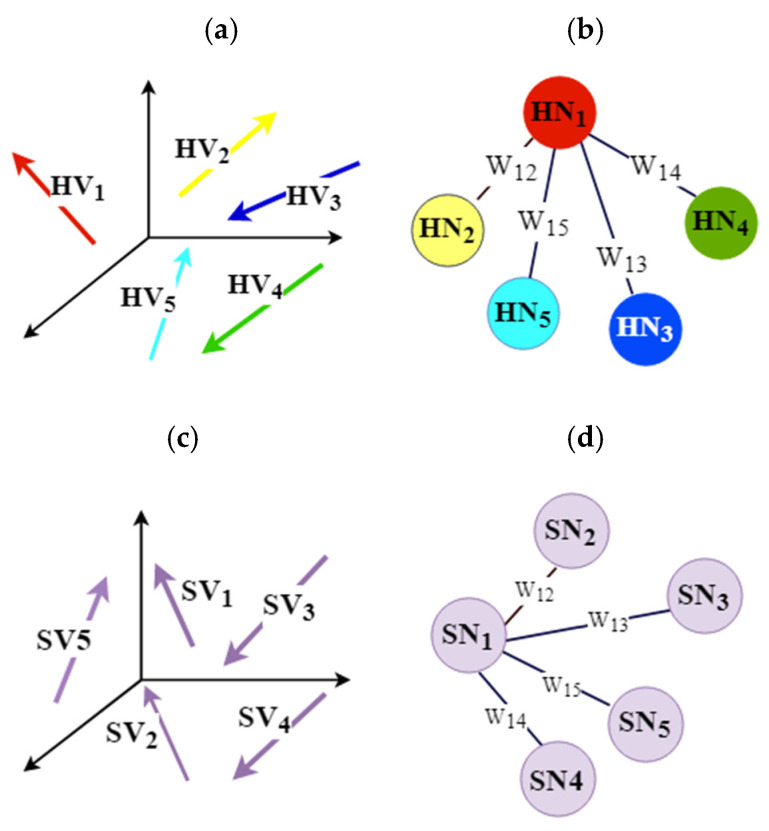
Transformation of 3D vectors into the weighted fully connected graph: (**a**) α-helix vectors in $R_{\mathrm{SSEs}-A}^{3}$; (**b**) construction of the weighted fully connected graph of α-helices ($G_{\mathrm{SSEs}-A}$ ). The *i*th helix vector (HV_i_) is transformed into an *i*th helix node (HN_i_); (**c**) stick vectors in $R_{\mathrm{SSEs}-C}^{3}$; (**d**) construction of the weighted fully connected graph of sticks ($G_{\mathrm{SSEs}-C}$.). The *i*th stick vector (SV_i_) is transformed into the *i*th stick node (SN_i_).

Figure 4 illustrates the transformation of the 3D vectors to the 3D graphs. For the sake of simplicity, only the relevant edges of one node in the weighted fully connected graphs are illustrated.

Let $A=\left(A_{1}{\;A}_{2}‚\;\ldots {‚\;A}_{m}\right)$ be a set of SSEs-A detected from the atomic structure and $C=\left(C_{1}{\;C}_{2}‚\;\ldots {‚\;C}_{n}\right)$ be a set of SSEs-C extracted on the cryo-EM map. The weighted fully connected graph of SSEs-A and SSEs-C are undirected fully connected graphs that are represented as a 4-tuple $G_{\mathrm{SSEs}-A}=\left(N_{A},{\;E}_{A},V_{A},W_{A}\right)$ and $G_{\mathrm{SSEs}-C}=\left(N_{C},{\;E}_{C},V_{C},W_{C}\right)$, respectively. Note that, since the process of construction of the $G_{\mathrm{SSEs}-A}$ and $G_{\mathrm{SSEs}-C}$ graphs are the same, for summarizing, the construction of the $G_{\mathrm{SSEs}-A}$ graph in the following has been elaborated.

Given $G_{\mathrm{SSEs}-A}=\left(N_{A},{\;E}_{A},V_{A},W_{A}\right)$, the first element of the $G_{\mathrm{SSEs}-A}$ graph is $N_{A}$, which is a nonempty set of nodes that represent the vectors of SSEs-A in the 3D space. *|*$N_{A}$*|* denotes the number of nodes, which is equal to the number of vectors in $R_{\mathrm{SSEs}-A\;}^{3}$. The second element of the graph is $E_{A}$, which is defined as a set of edges representing all possible interactions of nodes. The third element, $V_{A}$, is a set of labels of the nodes and they are defined based on the spatial position of $C_{\propto }$ atoms. It is appropriate to assign a pair $\left(s_{i}^{\to },\;e_{i}^{\to }\right)=\left(\langle x_{i}^{s},\;y_{i}^{s},\;z_{i}^{s}\rangle ,\langle x_{i}^{e},\;y_{i}^{e},\;z_{i}^{e}\rangle \right)$ from the start and end points of the *i*th vector to *i*th SSEs-A node of the graph. $s_{i}^{\to }$ and $e_{i}^{\to }$ are the first and the last $C_{\propto }$ coordinate voxels of the *i*th SSEs-A which is corresponded to the start and end voxel of the *i*th SSEs-A vector (${\mathrm{HV}}_{i}$.). The last element of the graph, $W_{A}$, is defined for assigning weights to the edges of the graph according to the mathematical-based features. More details about the construction of the three graphs based on the three mathematical-based features are provided as follows:

*i*. Angle-based fully connected graph *(*$G_{\mathrm{SSEs}-A}^{Angle}$.): This graph uses the angle of vectors for assigning weights to the edges of the graph. $W_{\mathrm{SSEs}-A}^{Angle}\left(e_{i}\;,e_{j}\right)$ is defined to calculate the weights of the $G_{\mathrm{SSEs}-A\;}^{Angle}$. graph based on the angle of every two vectors:
  (1) $$\;W_{\mathrm{SSEs}-A}^{Angle}\left(e_{i}\;,e_{j}\right)=\frac{\left(\;e_{i}^{\to }\;.e_{j}^{\to }\;\right)}{\|e_{i}^{\to }\|\;\|e_{j}^{\to }\|\;}\;,\;\forall \;e_{i}\;,e_{j}\;\epsilon \;HN_{i}\;.\;$$
*ii*. Euclidean distance-based fully connected graph ($G_{\mathrm{SSEs}-A}^{ED}$.): This graph utilizes the Euclidean distance (ED) metric for assigning weights to the edges of the $G_{\mathrm{SSEs}-A}^{ED}$. graph. The edge’s weight of the graph is computed based on the Euclidean distance of the midpoint of two vectors as follows:
  (2) $$m_{i}^{\to }=\frac{s_{i}^{\to }+e_{i}^{\to }\;}{2},\;m_{j}^{\to }=\frac{s_{j}^{\to }+e_{j}^{\to }\;}{2},\;W_{\mathrm{SSEs}-A}^{ED}\left(m_{i}\;,m_{j}\;\right)=\|m_{i}^{\to }-m_{j}^{\to }\|$$
*iii*. Relative length-based fully connected graph ($G_{\mathrm{SSEs}-A}^{RL}$.): This graph determines the weight of the edge based on the relative length (RL) of two vectors. This characteristic is defined to specify the relative length between two vectors and is computed based on Equation (3).
  (3) $$L_{i}=\left|s_{i}^{\to }-e_{i}^{\to }\right|,\;W_{\mathrm{SSEs}-A}^{RL}\left(L_{i},L_{j}\right)=\frac{\left|L_{i}-L_{j}\right|}{\left(L_{i}+L_{j}\right)}\;$$

According to the aforementioned three constructed graphs, three weighted adjacency matrices for $G_{\mathrm{SSEs}-A}$ have been constructed. Based on the same principle, three graphs and three weighted adjacency matrices for $G_{\mathrm{SSEs}-C}$. have been constructed. The $G_{\mathrm{SSEs}-A}$ and $G_{\mathrm{SSEs}-C}\;$ matrices are m×m and n×n, respectively. The characteristics of the matrices are:

- All entries on the main diagonal are zero ($x_{ii}$ = 0);
- All off-diagonal entries are positive ($x_{ij}$ > 0 if ***i ≠ j***);
- The matrices are a symmetric matrix (
  $$x_{ij}=x_{ji}$$).

In the following phase of the study, to compute the similarity of the nodes between the $G_{\mathrm{SSEs}-A}$. and $G_{\mathrm{SSEs}-C}$. graphs, two robust statistical scoring functions, BD and MAC, have been proposed. The Bhattacharyya distance (BD) computes the distance of two probability distributions or variables based on the statistical moments of the data [46]. These statistical indicators have been widely applied in signal processing [47], image processing [48], speaker recognition [49], and pattern recognition [50]. In this study, the metric is utilized to measure the geometrical similarity and to calculate the distance between all nodes of the $G_{\mathrm{SSEs}-A}$ and $G_{\mathrm{SSEs}-C}$ graphs. For more clarification, suppose that $r_{i}^{\mathrm{SSEs}-A\;}$ and $r_{j}^{\mathrm{SSEs}-C\;}$ are two rows of two weighted adjacency matrices. In detail, $r_{i}^{\mathrm{SSEs}-A\;}$ is the *i*th row of $Matrix_{\mathrm{SSEs}-A\;}$ and $r_{j}^{\mathrm{SSEs}-C}$ is the jth row of $Matrix_{\mathrm{SSEs}-C\;}$. $r_{i}^{\mathrm{SSEs}-A\;}$ signifies the weights of all adjacency edges for the *i*th SSEs-A node. Similarly, $r_{j}^{\mathrm{SSEs}-C}$ indicates the weights of all adjacency edges for the jth SSEs-C node. To compute the similarity score between the two nodes of $G_{\mathrm{SSEs}-A}$ and $G_{\mathrm{SSEs}-C}$, the following formula has been applied:

(4) $$BD\;\left(r_{i}^{\mathrm{SSEs}-A\;}\;,r_{j}^{\mathrm{SSEs}-C}\;\right)=-\ln\left(\sum^{\;}\sqrt{\left(r_{i}^{\mathrm{SSEs}-A\;}\;\right).\left(r_{j}^{\mathrm{SSEs}-C}\;\right)}\right),\;\forall \;i\;\epsilon \;1\leq i\leq \;m,\;\forall \;j\;\epsilon \;1\leq j\leq \;n$$

The calculated distance score (BD) determines the relative closeness of two nodes in two peer graphs. The BD scoring function varies between 0 to 1 (0≤BD≤1), in which BD=0 represents two nodes with high similarity, and vice versa. We applied the BD scoring function for all nodes of three peer graphs (i.e., $<G_{\mathrm{SSEs}-A\;}^{Angle},\;G_{\mathrm{SSEs}-C}^{Angle}>$, $<G_{\mathrm{SSEs}-A\;}^{ED},\;G_{\mathrm{SSEs}-C}^{ED}>$, $<G_{Helix}^{RL},\;G_{stick}^{RL}>$) to achieve the initial correspondence set for each pair of graphs. 

The second proposed scoring function, the modal assurance criterion (MAC), is a robust statistical metric that provides a measure of consistency between two linear arrays [51,52]. The basic idea behind the metric comes from the modal assurance criterion, which computes a measure of consistency between the experimental and the analytical modal arrays. In this study, the MAC considers as a scoring function to calculate the similarity of nodes in each two peer graphs based on Equation (5). Similar to the BD scoring function, the MAC metric takes two rows (i.e., $\;r_{i}^{\mathrm{SSEs}-A\;}$ and $r_{j}^{\mathrm{SSEs}-C}$.) of two peer matrices (e.g., $G_{\mathrm{SSEs}-A\;}^{Angle},\;G_{\mathrm{SSEs}-C}^{Angle}$) as inputs and computes the similarity score. The generated similarity score is in the range of 0–1, where a zero score indicates no consistency between the two peer nodes of the graphs, and one indicates complete consistency.

(5) $${\mathrm{MAC}}_{\mathrm{SSEs}-A,\mathrm{SSEs}-C}={\left(\frac{{\left({\left(r_{i}^{\mathrm{SSEs}-A\;}\;\right)}^{T}.\left(r_{j}^{\mathrm{SSEs}-C}\;\right)\right)}^{2}}{\left(\left({\left(r_{i}^{\mathrm{SSEs}-A\;}\;\right)}^{T}.r_{i}^{\mathrm{SSEs}-A\;}\;\right).\left({\left(r_{j}^{\mathrm{SSEs}-C}\;\right)}^{T}.r_{j}^{\mathrm{SSEs}-C}\;\right)\;\right)}\right)}_{i}\;$$

After applying the two aforementioned distance/similarity scoring functions on the three peer graphs, three candidate SSEs correspondence sets were generated. To extract the final SSEs correspondence among the three obtained candidate SSEs correspondence sets, a similarity-based voting algorithm has been developed. 

### 2.4. Similarity-Based Voting Algorithm (SimVA)

The similarity-based voting algorithm (SimVA) has been proposed as a decision-making strategy to extract the final SSEs correspondence among the three generated correspondence sets. The SimVA initially takes the three obtained correspondence sets as inputs and then generates the final SSEs correspondence as output. The final correspondences are extracted in three steps, including (i) unanimous voting, (ii) majority voting, and (iii) the principle of least conflict (PLC). These steps are presented in the following in detail.

#### 2.4.1. Unanimous Voting

In this step, the SimVA algorithm considers an assignment as an acceptable assignment if it is repeated in all the three candidate correspondence sets. In the other words, if *i*th SSEs-A matches with the jth SSEs-C based on the three mathematical-based features (angle, Euclidian distance, and relative length), this assignment is a great choice, and it is reported as an acceptable assignment.

#### 2.4.2. Majority Voting 

This routine supposes an assignment to be an acceptable assignment when it is repeated in the two candidate correspondence sets among the three correspondence sets. For example, if the *i*th SSEs-A match with the jth SSEs-C based on two of the mathematical-based features out of three, it is considered as an acceptable assignment and is inserted into the final correspondence set.

#### 2.4.3. Principle of Least Conflict

The main idea behind the principle of least conflict (PLC) approach is to find the assignments in the case that there is a remaining assignment that has not been selected in the two previous steps. In this step, the assignment with the minimum conflict has been recognized and selected as an acceptable assignment. The minimum conflict assignment is a <SSEs-A, SSEs-C> pair that has the least conflict with the other pairs. As an example, if the 1st SSEs-A should match with the 4th SSEs-C (i.e., the pair <1, 4> is a true assignment), all the other assignments except <1, 4> for the 1st SSEs-A (e.g., <1, 2>, <1, 3>, … <1, n>) are considered as conflict pairs. On the other hand, for the 4th SSEs-C, all other assignments except <1, 4> are also in conflict (e.g., <2, 4>, <3, 4>, … <m, 4>). After all the conflict pairs have been detected for all assignments, the number of conflict pairs for each assignment has been enumerated and the pair with the minimum number of conflicts is selected as an acceptable assignment. The proposed concept allows the SimVA algorithm to continue at times when we could not find the assignment from the two aforementioned voting routines in each iteration of the algorithm. At the end, all the acceptable assignments obtained from the SimVA algorithm are considered as a final SSEs correspondence. 

## 3. Results

This section presents experiments which have been designed to evaluate the robustness of the presented method. The effectiveness of the method was validated on 25 experimental and simulated cryo-EM maps in terms of precision, sensitivity, F-measure, and accuracy. The validity of the proposed approach was carried out by comparing the SSEs correspondence computed by the method presented in this study with the native correspondence (true SSEs correspondence). The native correspondence is obtained from the manual labeling of the SSEs in the density map based on the known atomic structure (for simulated data) or a structural homolog (for experimental data). We calculate the accuracy, precision, sensitivity, and F-measure based on the following formula:

(6)  Accuracy=TP+TN/(TP+FP+FN+TN)*100 

(7)  Precision=TP/(TP+FP)*100

(8)  Sensitivity=TP/(TP+FN)*100

(9) F−measure=(2×Precision×Sensitivity)/(Precision+Sensitivity)∗100

In the aforementioned equations, true positive (TP) is the number of detected matched SSEs that are correct, true negative (TN) represents the number of detected unmatched SSEs that are correct, false positive (FP) denotes the number of matched SSEs that are incorrect, and false negative (FN) is the number of rejected matched SSEs that are incorrect.

### 3.1. Experimental and Simulated Cryo-EM Density Maps

The efficiency and accuracy of the automatic method were tested using 25 α-β proteins. The data set of interest consists of 10 experimental and 15 simulated cryo-EM maps. The experimental cryo-EM maps, which are reported in Table 1, were obtained from the Electron Microscopy Data Bank (EMDB) [53] so that their resolutions ranges from 3.7 to 8.9 Å. 

The simulated maps, which are represented in Table 2, are synthesized at 10 Å resolution using the Chimera package [29], and the structure of the proteins were downloaded from the Protein Data Bank (PDB) (https://www.rcsb.org/, accessed on 30 September 2021) [54]. 

In the dataset of interest, the lengths of the proteins range from 117 (PDB ID: 3FIN) to 1703 (PDB ID: 6UXW) amino acid residues. The largest test case (PDB ID: 5KBU) in this dataset includes 65 SSEs-A and 54 SSEs-C. Therefore, the selected data set is appropriate to evaluate the robustness and effectiveness of the method in handling large samples. 

### 3.2. Performance Comparison of Two Scoring Functions

As described in the earlier section, three peer graphs from SSEs-A and SSEs-C (i.e., $<G_{\mathrm{SSEs}-A}^{Angle},\;\;G_{\mathrm{SSEs}-C}^{Angle}>$, $\;\langle G_{\mathrm{SSEs}-A}^{ED},\;\;G_{\mathrm{SSEs}-C}^{ED}\rangle$, $\;\langle G_{\mathrm{SSEs}-A}^{RL},\;\;G_{\mathrm{SSEs}-C}^{RL}\rangle$) have been constructed based on the three mathematical-based features. To measure the similarity of the nodes in each peer graph, two statistical scoring functions, BD and MAC, have been utilized. To assess the quality of the algorithm, we have evaluated our work based on the three proposed mathematical-based features using the BD and MAC scoring functions. The accuracy of the achieved SSEs correspondence sets (angle-, ED-, and RL-based correspondence sets) is calculated based on the Equation (6), as reported in Table 3. 

As can be seen in Table 3, the percentage of the average accuracy based on the angle-, ED-, and RL-based correspondence sets concerning the BD scoring function are equal to 53.20%, 69.39%, and 50.63%, respectively. For the MAC scoring function, these values are identical to 57.59%, 70.58%, and 53.76%, respectively. The results indicate that the MAC metric is more reliable than BD in finding the similarity of the nodes of the graphs.

To extract the final SSEs correspondence set from the three produced correspondence ones, the SimVA algorithm has been designed and implemented. In the following, the effectiveness of the developed algorithm is assessed on the experimental and simulated cryo-EM map. 

### 3.3. Impact of the SimVA Algorithm on the SSEs Correspondence Result

To improve the efficiency of the matching process, the SimVA algorithm has been proposed. The SimVA algorithm has been developed to extract the final SSEs correspondence based on the feature integration strategy. Here, the accuracy of the SimVA algorithm using two scoring functions, BD and MAC, is analyzed. Table 4 compares the performance of the method before and after incorporating the SimVA algorithm. 

A comparison of the reported results in Table 4 shows that for 24 out of 25 test cases, the accuracy has been improved by incorporating the SimVA algorithm. The total average accuracy obtained from the three mathematical-based features using BD and MAC is 57.74% and 61.51%, respectively. After incorporating the SimVA algorithm in the final step, the total average of the accuracy using BD and MAC are equal to 76.17 % and 76.09%, respectively. This reveals that incorporating the SimVA algorithm led to an 18.43% and a 14.58% improvement in the accuracy of the method. 

### 3.4. Assessment of the Method

**Figure 5:**
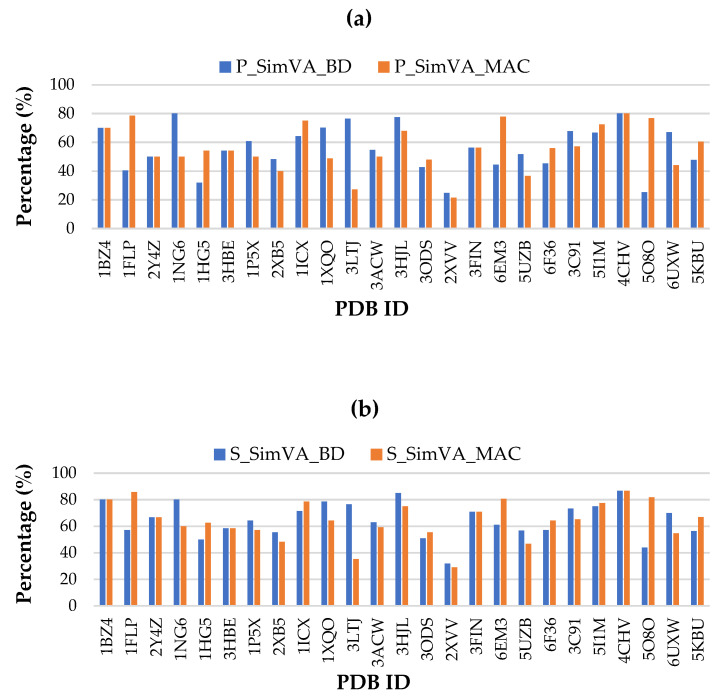

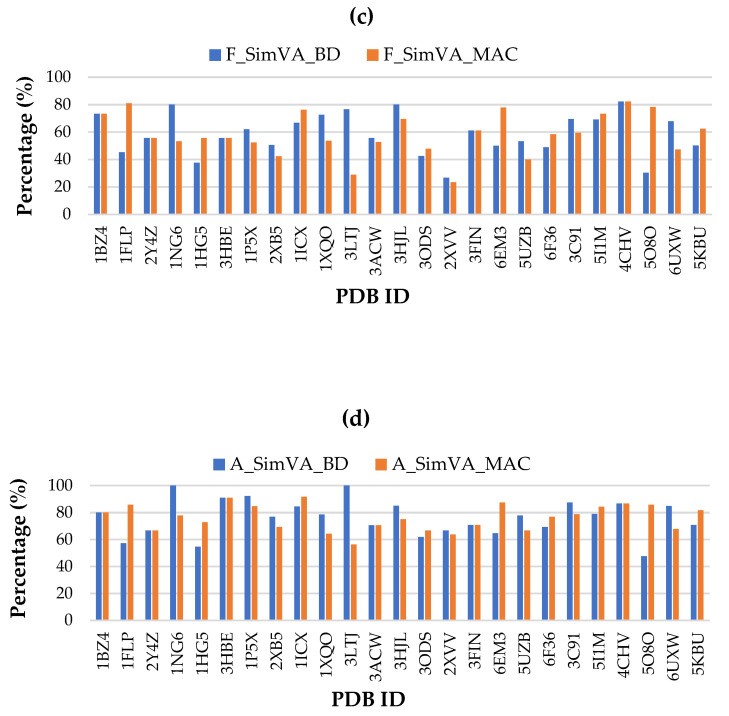
Assessment of the method concerning the performance measurements: (**a**) precision, (**b**) sensitivity, (**c**) F-measure, (**d**) accuracy.

To analyze the robustness of the method, four performance measurements (precision (P), sensitivity (S), F-measure (F), and accuracy (A)) were used. Figure 5 demonstrates the efficiency of the method using the measurements on the data set of interest. 

As can be observed in Figure 5, for most of the proteins in the data set with the aid of the SimVA_MAC, the accuracy is more than 70%. The results show that the method is robust and works well even under the presence of errors and uncertainties in the extracted SSEs in the cryo-EM images. This is a valuable outcome of this study.

### 3.5. Comparison of Method with DP-TOSS

In this section, the accuracy of the SimVA algorithm using two scoring functions, BD and MAC, has been compared with DP-TOSS [20]. Many approaches have recently been developed to solve the SSEs mapping problem for medium-resolution cryo-EM maps, as discussed in the introduction. Here, the proposed method is compared with the latest version of DP-TOSS. As can be seen in Table 5, the average of accuracy on the data set of interest for DP-TOSS, SimVA_BD, and SimVA_MAC are equal to 61.35%, 76.17%, and 76.09%, respectively. 

Based on the obtained results, it can be concluded that SimVA is more efficient than DP-TOSS. More specifically, the percentages of the accuracy improvement of the proposed method compared to DP-TOSS using the BD and MAC are equal to 14.82% and 14.74%, respectively. Furthermore, SimVA is able to work on large protein with a total number of 65 SSEs (PDB ID 5KBU). This is one of the valuable achievements of this study that can cope with the problem of using large complex proteins with many secondary structure elements. Working on large complex proteins has been a challenging issue in recent studies [18,19,20,54]. As reported in the state-of-the-art studies, the largest protein in their dataset includes 33 SSEs-A and 20 SSEs-C. In the current study, we have been able to run the designed automatic method on two experimentally huge cryo-EM maps, 6UXW (PDB ID) and 5KBU (PDB-ID), which consist of 1034 and 1703 amino acids, respectively. 

### 3.6. Runtime of the Method

**Figure 6:**
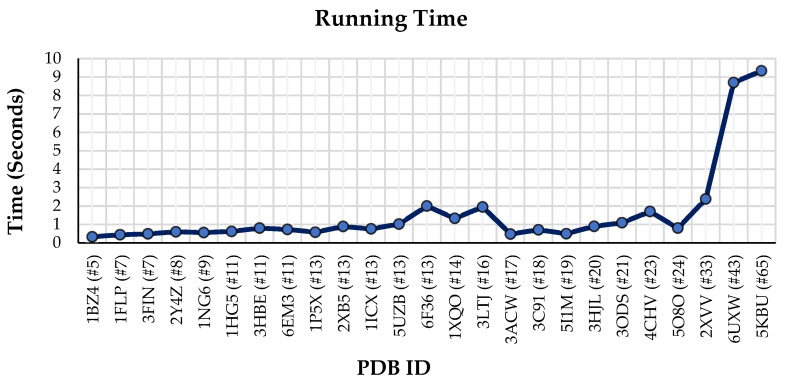
The runtime of the method with respect to the number of SSEs-A in proteins. (PDB ID (#SSEs-A)).

The proposed automatic matching algorithm consists of four main steps. The first step is to extract the SSEs from two sources of information (i.e., PDB and map), the second step is to construct the 3D vectors from extracted SSEs, the third step is to transform the 3D vectors into the 3D graphs, and the last step is to develop a similarity-based voting algorithm in order to obtain the final SSEs correspondence. Here, the runtime of the method has been computed for the last three steps. The total runtime has been computed on a workstation with MacBook Pro, 2.2 GHz 6-Core Intel Core i7 Processor, and 16 GB of memory. The running time of the method on the benchmark data set is illustrated in Figure 6. 

As can be observed in Figure 6, the runtime of the algorithm increases as the number of SSEs-A increases. For example, the least running time (0.46 s) is related to the protein 1BZ4 (PDB ID) with 5 SSEs-A, and the most running time (10.58 s) is relevant to the protein 5KBU (PDB ID) with 65 SSEs-A. 

## 4. Discussion and Conclusions

Cryo-EM has played an increasing role in the structure determination of molecular complexes in recent years. Despite many advances in cryo-EM technologies, in some cases, the resolution of the generated maps ranges between 4Å to 10Å. Therefore, the medium-resolution cryo-EM map may not be adequate to directly determine the atomic structure of the protein. At medium-resolution, the secondary structure elements have been extracted and visualized by various methods. In this study, the automatic assignment method has been developed to find the mapping of the secondary structures of the modeled structure to the cryo-EM map. Knowing this assignment allows us to form an initial hypothesis on the structure of the protein backbone. The key idea of the 3D matching strategy proposed in this study is to represent the extracted SSEs from the density map and the modeled structure in a common way, and then build up the correspondence between these two representations. Our common approach is 3D weighted fully connected graphs, with nodes representing the SSEs and the edges representing the connectivity between the SSEs. The key contributions of the geometrical matching method can be summarized as follows: (i) the modeling of the SSEs to the geometrical vectors in 3D space, (ii) transforming the 3D vectors into the 3D graphs based on the proposed mathematical-based features, (iii) introducing two robust statistical scoring functions, BD and MAC, to measure the similarity of nodes of the graphs, and (iv) developing the innovative similarity-based voting algorithm combined with the PLC concept to find the true correspondence. It is important to mention that the SSEs correspondence may not be a bijection. Due to the noise and uncertainty in a typical map, the SSEs detection algorithms may fail to find the location of all the SSEs within the map and may also identify false SSEs. We demonstrated the performance of the method on the simulated as well as experimental data sets in the presence of errors. Comparative studies have also been conducted to demonstrate the superiority of the 3D matching method over some of the existing state-of-the-art techniques. The results show that the automatic method is highly efficient (76.09% overall accuracy) and works well for large cryo-EM maps. Moreover, the key strength of the matching method is that it does not require any prior segmentation of the density map and does not need skeleton data to obtain the SSEs correspondence. Besides, the automatic method is able to work on the large cryo-EM data (PDB ID 5KBU) containing 65 SSEs-A and 54 SSEs-C with 81.62% accuracy in less than 11 s. 

## 5. Code Availability

The source code and data of the method is publicly available at https://github.com/Bahareh-Behkamal/Match_SSEs_CryoEM, accessed on 20 November 2021. Moreover, the instruction for utilizing the method can be found in the shared readme file.

## Figures and Tables

**Table 1:** The information of the experimental cryo-EM maps.

No	EMDB ID ^a^	PDB ID ^b^	Chain ^c^	# Length ^d^	# SSEs-A ^e^	# SSEs-C ^f^	Resolution ^g^
1	5030	3FIN *	R	117	7	7	6.4
2	3888	6EM3 *	A	291	11	9	4.2
3	8625	5UZB *	A	177	13	9	7
4	4176	6F36 *	M	327	13	11	3.7
5	1733	3C91 *	A	233	18	15	6.8
6	8070	5I1M *	V	458	19	17	7
7	2526	4CHV *	A	361	23	22	7
8	3761	5O8O *	A	349	24	22	6.8
9	20934	6UXW *	A	1703	43	35	8.9
10	8231	5KBU *	A	1034	65	54	7.8

^a^ The EMDB ID of the protein used in the test; ^b^ the PDB ID of the protein used in the test. β-containing proteins are marked with *; ^c^ the protein chain; ^d^ the number of amino acid residues in the sequence; ^e^ the total number of secondary structure elements (α-helices and β-strands) in the atomic structure; ^f^ the total number of secondary structure elements (α-helices and β-strands) extracted from the cryo-EM map; ^g^ the resolution of the experimental map in angstrom (Å).

**Table 2:** The information of the simulated cryo-EM map.

No	Name ^a^	PDB ID ^b^	Uniprot ID ^c^	Chain ^d^	Length ^e^	#SSEs-A ^f^	#SSEs-C ^g^
1	Apolipoprotein E	1BZ4	P02649	A	144	5	5
2	Hemoglobin-1	1FLP	P41260	A	142	7	7
3	Gag polyprotein	2Y4Z *	P03336	A	140	8	8
4	Uncharacterized protein YqeY	1NG6	P54464	A	148	9	7
5	Phosphatidylinositol	1HG5	O55012	A	289	11	9
6	Class IV chitinase Chia4-Pa2	3HBE	Q6WSR8	X	204	11	7
7	Phospholipase C	1P5X	P09598	A	245	13	9
8	Tetracycline repressor protein class D	2XB5	P0ACT4	A	207	13	9
9	Protein LlR18A	1ICX *	P52778	A	155	13	11
10	N-glycosylase/DNA lyase	1XQO	Q8ZVK6	A	256	14	14
11	AlphaRep-4	3LTJ	__	A	201	16	12
12	4,4’-diapophytoene synthases	3ACW	A9JQL9	A	293	17	14
13	Flagellar motor switch protein FliG	3HJL	O66891	A	329	20	20
14	Symplekin	3ODS	Q92797	A	415	21	16
15	Albumin	2XVV	P02768	A	585	33	19

^a^ the name of the protein; ^b^ the PDB ID of the protein used in the test. β-containing proteins are marked with *; ^c^ the Uniport ID of the protein; ^d^ the protein chain; ^e^ the number of amino acid residues in the sequence; ^f^ the total number of secondary structure elements (α-helices and β-strands) in the atomic structure; ^g^ the total number of secondary structure elements extracted from the cryo-EM map.

**Table 3:** The accuracy of the three SSEs correspondence sets using two scoring functions.

			BD			MAC	
NO	PDB ID	Angle	ED	RL	Angle	ED	RL
1	1BZ4	80	80	80	80	60	80
2	1FLP	42.85	57.14	28.57	57.14	71.42	57.14
3	2Y4Z	50	58.33	58.33	58.33	50	50
4	1NG6	44.44	88.88	66.66	44.44	88.88	77.77
5	1HG5	72.72	36.36	36.36	54.54	45.45	54.54
6	3HBE	81.81	90.9	81.81	81.81	90.9	72.72
7	1P5X	69.23	84.16	61.53	76.92	100	69.23
8	2XB5	38.46	76.92	69.23	46.15	53.84	69.23
9	1ICX	76.19	77.38	53.57	84.52	70.23	63.09
10	1XQO	64.28	57.14	50	71.42	78.57	28.57
11	3LTJ	43.75	93.75	37.5	100	43.75	62.5
12	3ACW	35.29	64.7	47.05	35.29	52.94	35.29
13	3HJL	20	90	30	40	95	30
14	3ODS	33.33	52.38	33.33	23.8	57.14	42.58
15	2XVV	60.6	78.78	45.45	63.63	78.78	54.54
16	3FIN	58.33	58.33	29.16	45.83	87.5	58.33
17	6EM3	70.83	47.91	58.33	81.25	54.16	52.08
18	5UZB	55.55	66.66	44.44	55.55	66.66	55.55
19	6F36	38.46	92.3	53.84	38.46	100	53.84
20	3C91	62.5	63.75	60	62.5	68.75	45
21	5I1M	36.84	52.63	57.89	31.57	47.36	36.84
22	4CHV	53.33	73.33	46.66	53.33	93.33	66.66
23	5O8O	52.38	66.66	52.38	50	92.85	50
24	6UXW	41.21	79.84	48.18	49.69	67.27	41.66
25	5KBU	47.63	46.59	35.51	53.78	49.76	36.97
	Average	53.20	69.39	50.63	57.59	70.58	53.76

**Table 4:** The accuracy of the method incorporating the SimVA algorithm.

No	PDB ID ^a^	BD ^b^	SimVA_BD ^c^	MAC ^d^	SimVA_MAC ^e^
1	1BZ4	80	80	73.33	80
2	1FLP	42.85	57.14	61.9	85.71
3	2Y4Z	55.55	66.66	55.55	66.66
4	1NG6	66.66	100	70.37	77.77
5	1HG5	48.48	54.54	51.51	72.72
6	3HBE	84.84	90.9	81.81	90.9
7	1P5X	71.79	92.3	82.05	84.61
8	2XB5	61.53	76.92	56.4	69.23
9	1ICX	69.04	84.52	72.61	91.66
10	1XQO	57.14	78.57	59.52	64.28
11	3LTJ	58.33	100	62.5	56.25
12	3ACW	49.01	70.58	41.17	70.58
13	3HJL	46.66	85	55	75
14	3ODS	39.68	61.9	41.26	66.66
15	2XVV	61.61	66.66	65.65	63.63
16	3FIN	48.61	70.83	63.88	70.83
17	6EM3	59.02	64.58	62.5	87.5
18	5UZB	55.55	77.77	59.25	66.66
19	6F36	61.53	69.23	64.1	76.92
20	3C91	62.08	87.5	58.75	78.75
21	5I1M	49.12	78.94	38.59	84.21
22	4CHV	57.77	86.66	71.11	86.66
23	5O8O	57.14	47.61	64.28	85.71
24	6UXW	56.41	84.84	52.87	67.87
25	5KBU	43.24	70.73	46.84	81.62
	Average	57.74	76.17	61.51	76.09

^a^ the PDB ID of the protein; ^b^ the total accuracy obtained from three mathematical-based features using BD scoring function; ^c^ the accuracy of the SimVA algorithm using BD scoring function; ^d^ the total accuracy obtained from three mathematical-based features using MAC scoring function. ^e^ the accuracy of the SimVA algorithm using the MAC scoring function.

**Table 5:** Comparison between DP-TOSS and SimVA.

No	PDB ID ^a^	DP-TOSS ^b^	SimVA_BD ^c^	SimVA_MAC ^d^
1	1BZ4	100	80	80
2	1FLP	100	57.14	85.71
3	2Y4Z	50	66.66	66.66
4	1NG6	71.40	100	77.77
5	1HG5	55.60	54.54	72.72
6	3HBE	57.10	90.9	90.9
7	1P5X	55.60	92.3	84.61
8	2XB5	66.70	76.92	69.23
9	1ICX	45.50	84.52	91.66
10	1XQO	71.4	78.57	64.28
11	3LTJ	83.30	100	56.25
12	3ACW	100	70.58	70.58
13	3HJL	100	85	75
14	3ODS	100	61.9	66.66
15	2XVV	89.40	66.66	63.63
16	3FIN	100	70.83	70.83
17	6EM3	44.40	64.58	87.5
18	5UZB	55.50	77.77	66.66
19	6F36	100	69.23	76.92
20	3C91	46.70	87.5	78.75
21	5I1M	41.20	78.94	84.21
22	4CHV	0	86.66	86.66
23	5O8O	0	47.61	85.71
24	6UXW	0	84.84	67.87
25	5KBU	0	70.73	81.62
	Average	61.35	76.17	76.09

^a^ the PDB ID of the protein; ^b^ the accuracy of DP-TOSS method; ^c^ the accuracy of the SimVA algorithm using BD scoring function; ^d^ the accuracy of the SimVA algorithm using MAC scoring function.
